# Possible Selves: Implications for Psychotherapy

**DOI:** 10.1007/s11469-015-9553-2

**Published:** 2015-05-02

**Authors:** Waclaw Bak

**Affiliations:** Institute of Psychology, The John Paul II Catholic University of Lublin, Al. Raclawickie 14, 20-950 Lublin, Poland

**Keywords:** Possible selves, Self-standards, Psychotherapy, Self-regulation, Depression

## Abstract

The paper is devoted to the therapeutic applications of theories and research concerning self-regulation issues. The key concept here is possible selves, defined as an element of self-knowledge that refers to what a person perceives as potentially possible. The main idea of using knowledge about possible selves in psychotherapy is based on their functions as standards in self-regulatory processes. The problem of the changeability of possible selves and self-standards is analyzed in the context of their role in behavior change. The paper also presents the assumptions of Self-System Therapy – a newly developed cognitive therapy for depression, drawing directly on self-regulation theory and research.

Psychotherapists representing various approaches regard the patient’s possibilities as an important instrument of effecting a change. These possibilities are usually understood as a kind of positive resource that the patient can draw on when making desirable changes in his or her behavior (González et al. 2011; Kita 2011; O’Hanlon 1998; Yapko 1988; Zeig 2013). In the present article I draw on this idea, but I formulate a slightly broader perspective of looking at the client’s imaginations of his or her own possibilities (i.e., the possible future states of the self) and at the implications of those imaginations for psychotherapy. I refer to the concept of possible selves as one of the major concepts in the contemporary cognitive theories of self-knowledge and self-regulation. It is understood not only as a kind of positive resource facilitating change but also as a cognitive representation of the ultimate goal of that change.

The main idea of the article is that possible selves, being cognitive representations of alternative versions of the self, underlie so-called self-standards, which in turn constitute an important element of self-regulation processes. Research on self-regulation processes may be a source of compelling hypotheses concerning the pathogenesis of disorders as well as a source of ideas for innovative therapeutic interventions based on the achievements of cognitive personality psychology. In this context, I will analyze the problem of possible selves and the role they play in behavior change. I will also refer to one of the new forms of cognitive therapy for depression, which draws directly on self-regulation theory and research.

## The Possible Selves Theory

Self-knowledge comprises not only beliefs concerning the current state of the self (called the actual self) but also ideas and expectations concerning various potential states. The latter aspect of self-knowledge has been described in the possible selves theory by Markus and Nurius (1986). Although the possible selves theory does not explicitly address the problems of psychotherapy, the results of many studies inspired by it may be a source of interesting ideas concerning change in the course of psychotherapy.

According to Markus and Nurius’s (1986) classic approach, possible selves are a “future-projected” aspects of self-knowledge, which refers to what a person perceives as potentially possible with regard to himself or herself. Like all self-knowledge, possible selves are largely based on past experiences, but their essence lies in clear references to the future. They may be said to be imagined visions of oneself in the future (Erikson 2007; Hoyle and Sherrill 2006). As such, they are cognitive representations of hopes, fears, and fantasies regarding oneself.

It must be stressed that even though the term “possibilities” appears to have clearly positive connotations (having associations with positive resources that can be “used” beneficially), in the context of the theory referred to here such one-sided evaluation would oversimplify the matter. Possible selves are imagined future states of the self, and ideas of the future are not always positive. Thus, the concept of possible selves covers references not only to states that are positively evaluated and anticipated with hope but also to those possibilities that one would like to avoid, perceiving them as potentially threatening. The former are termed hoped-for selves and the latter – feared selves (Markus and Nurius 1986).

It is also worth noting that the plural is used in the concept of possible selves, clearly indicating that we are not dealing with a single possible self but with a multielement set of perceived possibilities. A person may generate many alternative versions of the self, either relating to different life domains or within one domain. These are not representations of some abstract personality traits or generic categories but comprehensive ideas of oneself in particular roles and situations.

## The Functions of Possible Selves

Markus and Nurius (1986) describe two important functions that possible selves perform in personality. Firstly, they constitute the context for evaluating the actual self and thus they are an important element of self-evaluation processes. The subjective evaluation of the current state of the self must take into account some point of reference – a certain potential state that is a criterion in evaluating the current state. Secondly, possible selves play an important role in motivational processes. They determine the direction of change and motivate the person to take action in order to realize the hoped-for visions of the self and to prevent the realization of the feared ones. Theoretically, any possible self can perform these motivational functions, but real influence on behavior is the most probable in the case of such possible selves that constitute cognitive representations of goals that are important for the self, which have been described in detail and, additionally, include specific behavioral strategies of achieving those goals.

To emphasize those motivational functions, the term *self-regulatory possible selves* is used in the literature as distinct from *self-enhancing possible selves* (Hoyle and Sherrill 2006; Oyserman et al. 2004). The latter type of possible selves mainly serves to reinforce self-esteem, optimism, and hope about the future but has no direct influence on behavior. The possible selves that mainly perform these emotional functions and do not clearly translate into motivation to change are usually formulated at a higher level of abstraction; they tend to be imprecise and not very specific. They also do not contain descriptions of action strategies aimed at achieving the goal. In order to perform regulatory functions effectively, possible selves should be formulated as specifically as possible and their content should relate to strategies of achieving the hoped-for state or avoiding the feared state of the self. Another important factor is the belief that a given possibility is achievable as well as a broader belief in the controllability of one’s own life. Moreover, in order to perform regulatory functions, possible selves should be integrated with the remaining elements of self-knowledge and should not come into conflict with significant aspects of identity (Oyserman and James 2009).

## Implications for Psychotherapy

The “future-projection” emphasized above and the regulatory functions of possible selves suggest potential implications for psychotherapy. If possible selves influence psychological well-being (involvement in self-evaluation processes) and play an important role in behavior change (the function of standards in self-regulation processes), then it seems reasonable to consider their role in the process of psychotherapy. Assuming that the essence of psychotherapy is change, the key issue for the present reflections will be the changeability vs. stability of possible selves. Let us, then, consider to what extent and on what conditions possible selves undergo changes and whether those changes occur in the course of psychotherapy.

The issue of whether possible selves are prone to change is not clear. In longitudinal research conducted over 5 years in a group aged from 55 to 89 years, relatively high stability of possible selves was observed (Frazier et al. 2000). On the other hand, Strauss and Goldberg (1999) demonstrated that a change in the role a person performs may significantly change the repertoire of possible selves. This research was conducted in a group of men who had entered the new role of the father with the birth of their first child. Such a change led to new possible selves being generated. What is more, the change promoted observable behavioral commitment to looking after the child. This result is consistent with the theory proposed by Markus and Nurius (1986), stressing the situation-dependence of the content of possible selves. A change of situation stimulates changes in possible selves, which in turn facilitates adaptation to the new situation.

It can therefore be said that although possible selves as such are fairly stable elements of self-knowledge (particularly in people above 50 years of age), a change of the situational context, as it were, imposes a different perspective of looking at oneself. Such a change in the repertoire of possible selves happens spontaneously, provided that there has been a sufficiently significant change of situation. It seems, however, that the modification of possible selves may also be an effect of more planned and intentional interventions – also as an element of psychotherapy. Encouraging the client to enter new and previously unknown context broadens his or her repertoire of possible visions of themselves and may promote change. Similar effects can probably be achieved using imaginative techniques (Lazarus 1984), consisting, for example, in mental preparation for performing a new role in life. The new context generates alternative versions of the self, which probably help in adapting to the new situation.

This effect was confirmed in the research conducted by Dunkel et al. (2006), which directly concerned changes in the repertoire of possible selves in the course of psychotherapy. These researchers referred to Prochaska’s model, which assumes that change in the course of psychotherapy is a process consisting of the following five stages: precontemplation, contemplation, decision or preparation, action, and maintenance (Prochaska and Norcross 2003). The results obtained by Dunkel et al. (2006) suggest that the precontemplation stage involves the smallest number of possible selves. This is the stage when a person does not have an intention to change, does not realize the existence of the problem, and avoids subjects related to it. In order to maintain this defensive state of unawareness, the person must avoid focusing on themselves – which results, among other things, in a limited repertoire of possible selves. However, in the very next stage – the contemplation stage – the person begins to be aware of the existence of the problem and consciously reflects on the possibilities of solving it. This, by definition, involves a greater exploration of the self, resulting in an increasing number of diverse possible selves. The study by Dunkel et al. (2006) referred to here showed that the contemplation stage does indeed correlate positively with the number of possible selves generated by a person. A similar situation occurs in the decision stage, when the repertoire of possible selves is the richest. This is because the decision-making process involves reflection on and imaginative processing of various possibilities, sometimes very different from one another. However, immediately after the decision has been made the number of possible selves decreases and in the following stages – action and maintenance – it is already smaller (though still higher than in the precontemplation stage). The realization of one of the planned options always entails abandoning the alternative options. As a result, the possible selves depicting the abandoned alternatives grow weaker and gradually disappear. In the next stage, the number of possible selves continues to decrease. At this stage, the person tries to maintain the change made and experiences that the reality initially perceived only as a potentiality has become a fact. It can be said that a possible self has materialized and its content has become part of the actual self. Meanwhile, a new possible self can be generated that will promote a new change and the person’s further development.

## Possible Selves as Standards in Self-Regulation Processes

The above reflections suggest potential applications of knowledge about possible selves in planning therapeutic interventions. In order to pursue this issue further, let us look more closely at the motivational and regulatory functions of possible selves. As mentioned above, one of the main functions that possible selves perform in personality is precisely their role in self-regulation processes. This issue has recently been an object of intensive research, whose results enable a more detailed description of this phenomenon and the principles governing it.

VanDellen and Hoyle (2008) mention two possible mechanisms by means of which it is possible to effect behavior change. The first one consists in increasing the cognitive availability of specific behavioral responses. If a possible self comprises not only a description of the end-state but also a kind of “recipe” for behavior that is supposed to lead to that state, then the activation of such a possible self increases the cognitive accessibility and attractiveness of particular responses and behaviors, which promotes change. The person engages in behaviors that contribute towards a positive change because the accessibility of cognitive representations of these behaviors is higher. The possible self works as a kind of know-how for a specific goal. This process takes place independently of comparisons with the actual self, which are the essence of the second mechanism of behavior change. This other mechanism is based on the evaluation of the actual self, in which the possible self plays the role of a criterion – or, in other words, a self-standard. The result of the evaluation of the actual self in the context of the standard sets off the self-regulation process.

Nowadays, many researchers emphasize that one of the central features of possible selves is precisely their function of standards in the processes of self-regulation and behavior change. Adopting such a perspective allows to place the concept of possible selves in the broader context of classic self-regulation theories, according to which a change of the current state of the self is motivated by the perceived discrepancy between the actual self and a particular self-standard (Carver and Scheier 1998; Duval and Wicklund 1972; Higgins 1997; Hoyle and Sherrill 2006).

The general idea of this approach was formulated by Duval and Wicklund (1972) as Objective Self-Awareness Theory (see also Silvia and Duval 2001). This theory assumes that focusing on oneself almost automatically triggers off the process of comparing the current state of the self with a specific standard appropriate to the situation. If, as a result of this comparison a person can see that he or she is the kind of person that the standard requires, they experience positive emotions, which motivate them to remain in the pleasant state of self-awareness. If, by contrast, the comparison reveals a significant discrepancy between the self and the standard, the sense of not meeting the standard generates negative emotions. This aversive emotional state motivates the person to reduce the discrepancy between the actual self and the standard or to escape from self-awareness when changing the discrepancy is judged to be too difficult.

Developing this idea, Carver and Scheier (1998) distinguished two types of standards and two corresponding self-regulatory systems. If a standard describes a certain desirable state of affairs, the self-regulation process consists in minimizing the perceived discrepancy between the self and the standard – striving to comply with the standard. If a standard has a negative form and refers to a certain undesirable vision that is to be avoided (Ogilvie 1987), self-regulation consists in maximizing the discrepancy between the self and the standard. A different classification was proposed by Higgins (1987), who distinguished between ideal and ought standards; these may be treated as more specific types of positive standards (Bak 2014). Regardless of specific differences between these theories, their essence lies in recognizing that the relationship between the actual self and a particular self-standard generates emotions and motivates to change.

The results of many studies show that discrepancies between the actual self and self-standards are linked to psychological disorders. This concerns many different forms of disorders, but it is especially important in the case of mood disorders and anxiety disorders (Bentall et al. 2005; Scott and O’Hara 1993; Strauman 1989). If we assume that one of the factors behind the appearance of problems with mental health is the discrepancy between the self and a standard, then the question arises of whether it is possible to modify this discrepancy. When the discrepancy is too large, its reduction can be effected either by changing the current self in such a way as to bring it closer to the standard or by modifying the content of the self-standard. This idea is referred to in one of the latest proposals of depression therapy, known as Self-System Therapy (Vieth et al. 2003), which the next section will be devoted to.

## Self-System Therapy

Self-System Therapy (SST) is a proposal of therapeutic intervention aimed at people whose depression problems stem from ineffective self-regulation (Vieth et al. 2003). The theoretical basis of SST is Higgins’s (1987) Self-Discrepancy Theory, describing the relations between the structure of the self and emotions, as well as a somewhat later theory by the same author – Regulatory Focus Theory (Higgins 1997), describing the promotion-focused vs. prevention-focused self-regulatory styles. The originators of SST assume that one of the major sources of depression is chronic failures – repeated or individual but concerning very important life domains – in achieving desirable states of affairs. In the language of Higgins’s (1987, 1997) theory, this means those aspects of self-regulation that are realized using promotion-focused strategies – as opposed to prevention-focused ones, which govern the avoidance of undesirable states.

Adopting such conceptualization of the sources of depression means assuming that an improvement in the effectiveness of self-regulation should lead to a reduction in the intensity of depressive symptoms. The authors do not say that the mechanism of pathogenesis they postulate operates in every case of depression. What they do emphasize is that the proposed new form of therapy is aimed at people whose depression stems from a strong promotion-focused orientation in self-regulation accompanied by a large discrepancy between the actual self and the ideal self (Vieth et al. 2003). Strauman et al. (2006) compared the effects of SST with the classic cognitive therapy of depression. It was found that even though the two forms of therapy did not differ in terms of overall effectiveness, SST was more effective with regard to those individuals whose depression stemmed from self-regulation problems.

Assuming that what lies at the root of problems with promotion-focused self-regulation strategies is a large actual-ideal self-discrepancy, it is concluded that change can be effected by means of the following three mechanisms: (1) modifying the content of self-knowledge by introducing new elements into it; (2) modifying the cognitive accessibility of particular elements of self-knowledge – increasing the accessibility of adaptive beliefs and reducing the accessibility of those beliefs that are constituents of the large actual-ideal self-discrepancy; (3) changing the perceived significance and/or consequences of particular self-beliefs.

Specific SST therapeutic techniques are similar in many respects to the techniques used in the classic cognitive therapy of depression or in interpersonal therapy. However, they are aimed at changing the maladaptive patterns of self-discrepancy and at “rebuilding” promotion-focused self-regulation strategies. An important technique is the analysis of interpersonal relations, in which, by experiencing the consequences of certain behaviors (or absence of behaviors), the patient learned what it meant to be a good person or a bad one. However, what is specific to SST is that interpersonal relations are not analyzed for their own sake but constitute the basis for the analysis of the patient’s predominant self-regulatory style. Another important element of therapy is the analysis of self-beliefs – their sources, contents, and functions, including the role they play in the development and persistence of depression symptoms (Vieth et al. 2003).

Self-System Therapy is a short-term (about 20–25 sessions) structured form of therapy comprising three phases: orientation, exploration, and change (transformation). The orientation phase takes up the first 5–7 sessions; its aim is to gain knowledge about depression and its therapy, to name one’s own depression problems, to begin discovering the relations between self-regulation problems and depression episodes, and to make attempts at more effective self-regulation. The second phase – exploration – takes up the next 8–12 sessions and is devoted to exploring two critical aspects of the patient’s self-regulation: his or her goals and regulatory style. The transformation phase usually takes up the last 8 sessions, which are focused on helping the patient develop more effective strategies of achieving goals (desired states of affairs). This happens through changing the maladaptive aspects of self-knowledge and self-regulation and through compensating for those maladaptive aspects that are resistant to change (Vieth et al. 2003).

## The Changeability of Self-Standards

The above reflections point to the special significance of self-standards in the etiology and therapy of depression. In accordance with the idea outlined here, one of the most important sources of depression is not so much the fact that a person fails to live up to his or her standards to a sufficient degree as the fact that those standards are unachievable in the first place. In such a situation, encouraging the person to make more efforts towards achieving the desirable state would not only be ineffective but would even intensify the symptoms of depression. It would be more reasonable in this situation to try to revise and modify the standard itself. At this point, a question arises of whether and in what conditions this kind of change is possible.

In the classic version of Objective Self-Awareness Theory, self-standards were treated as relatively stable (Duval and Wicklund 1972). In a situation of a large perceived discrepancy between the actual self and a self-standard, the person experiences emotional discomfort, which motivates them to make attempts to reduce the discrepancy. Theoretically, such a reduction can be achieved in two ways: (1) by changing behavior in such a way as to change the actual self and make it comply with the requirements of the standard or (2) by changing the standard towards adjusting it to the actual self. Initially, it was believed that, of these two theoretically possible ways, a change of behavior was more probable than a change of the standard. It was believed that a standard was a representation of a certain external norm and that, as such, it was less prone to change. However, subsequent research indicates that a change of standard is possible, although its introduction is less intuitive and requires meeting certain conditions as well as using a more intentional strategy.

In a situation of perceived discrepancy between the self and the standard, the natural tendency is to focus on the self and one’s own inadequate behavior. The standard constitutes a certain context – to use the language of Gestalt psychology, it is the “background” for the perception of the clearly distinct “figure” of the self. A person’s attention is focused on his or her own behavior evaluated against the standard. The standard itself, being the background and the context, is not in the center of attention and is thus not very likely to be spontaneously subjected to in-depth analysis and reflection. Consequently, a modification of the standard requires overcoming the habitual tendency to focus on the self, which means intentionally turning one’s attention away from the unsatisfactory present state and focusing on the standard itself (Duval and Lalwani 1999; Silvia and Duval 2001). This is a prerequisite of setting off the analysis of the standard’s contents and subjecting those contents to critical reflection, which may result in a modification of the standard. In such conditions, the person may, for example, conclude that the expectations they have of themselves are irrational or even harmful, which may induce him or her to amend the contents of those expectations.

## Conclusions

The above reflections indicate that appealing to the client’s beliefs concerning the potential, not yet realized versions of the self, referred to in cognitive psychology as possible selves, is potentially useful in the process of psychotherapy. Not to question the approach, where possibilities are understood as positive resources reinforcing motivation to change, the approach presented here stresses that one of the most important functions of possible selves is playing the role of standards in self-regulation. A possible self is a visualization of a goal that a person strives to achieve or of an anti-goal that he or she wants to avoid. The approach presented here, drawing on the theories and studies carried out in cognitive personality psychology, inspired an interesting proposal of a therapy, referred to as Self-System Therapy.
